# The complete mitochondrial genome of a freshwater mussel *Nodularia douglasiae* (Bivalvia: Unionidae)

**DOI:** 10.1080/23802359.2018.1471365

**Published:** 2018-08-17

**Authors:** Seung Hyun Cha, Jin Hee Lee, Eun Hwa Choi, Kuem Hee Jang, Young Jin Lim, Sang Gi Kim, Shi Hyun Ryu, Young Sup Lee, Ui Wook Hwang

**Affiliations:** a Department of Biology Education, Teachers College & Institute for Phylogenomics and Evolution, Kyungpook National University, Daegu, Republic of Korea;; b School of Life Sciences, Graduate School, Kyungpook National University, Daegu, Republic of Korea;; c Freshwater Biodiversity Research Division, Nakdonggang National Institute of Biological Resources, Sangju, Republic of Korea

**Keywords:** *Nodularia douglasiae*, complete mitochondrial genome, molecular phylogeny, Bivalvia

## Abstract

The circular F-type mitochondrial genome (15,761 bp) was completely sequenced for a Korean freshwater mussel *Nodularia douglasiae* (synonym *Unio douglasiae*; Unionidae, Unionida, Bivalvia). It contains 13 PCGs, two rRNA genes, and 22 tRNA genes, as generally shown in metazoan mitochondrial genomes. Its gene order is identical to that of F-type mitochondrial genomes observed in other freshwater mussels. With nucleotide and amino acid sequences of the complete F-type mitochondrial genomes obtained from 38 unionid species reported so far, phylogenetic analyses were done and discussed. The present study may give valuable helps to explore genetic diversity and population structures of other freshwater mussels as well as *N. douglasiae*.

Although *Nodularia douglasiae* (synonym *Unio douglasiae*; Unionidae, Unionida, Bivalvia) is one of the most common and widespread freshwater mussels in the whole river systems from Republic of Korea, and also widely and abundantly distributed in China, eastern Russia, and Japan (Liu et al. 2017), it has been considered as ‘Least Concern’ in the last IUCN Red List assessment (Allen and Bogan 2014). Recently, Wang et al. (2015) has first reported the complete mitochondrial genome of *N. douglasiae* from China, and Liu et al. (2017) has examined phylogenetic relationships and population structure of *N. douglasiae* from eastern Asia with partial COI genes.

Here, total cellular DNA was extracted from the fresh foot tissues of a female adult of *N. douglasiae* which was collected from the Han River system (Bangok-ri, Hongcheon-Gun, Gangwon-Do, Republic of Korea) and deposited at Institute for Phylogenomics and Evolution, Kyungpook National University, South Korea (voucher MoBiUn_001). The Korean F-type mitochondrial genome of *N. douglasiae* was completely sequenced, which is 15,761 bp long (GenBank accession number MF314443). It contains 13 PCGs, two rRNA genes (*12S* and *16S*), and 22 tRNA genes. The 22 tRNAs were predicted using the computer softwares of tRNAscan-SE 2.0 (Lowe and Chan 2016) and ARWEN (Laslett and Canbäck 2008). The results showed that the overall base composition of the *N*. *douglasiae* mitochondrial genome is 38.35% A, 26.53% T, 11.88% G, and 23.23% C. Its gene order was identical to those of *N. douglasiae* from China (Wang et al. 2015) and other F-type freshwater mussels reported so far. The total length of the gene encoding 13 proteins was 11,115 bp, accounting for 70.52% of the entire mitochondrial genome sequence. Three genes of *ND4*, *ND5*, and ND6 started with ATT codon, four genes of *CO1*, *CYTB*, *ND1*, and *ND4L* with ATA, and the remaining genes with ATG. All PCGs ended with one of the two canonical stop codons of TAA and TAG. There were relatively two large noncoding regions between *trnE* and *ND2* (319 bp) and between *ND5* and *trnQ* (290 bp). The locations of the noncoding regions were similar to those of *N. douglasiae* from China, but Korean ones were a little longer than those of China (312 and 275 bp, respectively).

The phylogenetic analyses were performed based on concatenated nucleotide sequence or amino acid sequence alignments of 12 PCGs (except for *ATP8*) and two rRNA genes from 38 unionids, using RAxML v8.0.0 (Stamatakis 2014) and MrBayes v3.2.1 (Ronquist et al. 2012). The results shown in Figure 1 indicated that *N. douglasiae* from Korea and China formed a monoclade, but the monophyly of Unioninae was not supported due to unexpected placements of the two unionids *Lanceolaria grayana* and *Lepidodesma languilati*. Except for Unioninae, the three remaining unionid subfamilies formed strong monophylies. This study would provide valuable information for understanding the genetic diversity of *N. douglasiae* and phylogenetic relationships among unionids.

**Figure 1.**
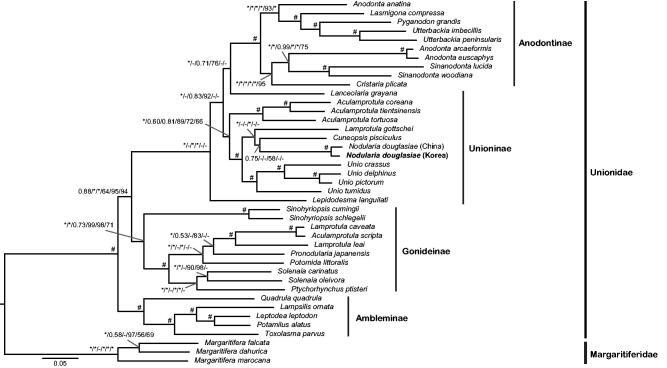
Bayesian inference tree of 38 unionids inferred from 12 PCGs and two rRNA genes, showing phylogenetic relationships among the four unionid subfamilies and phylogenetic position of *Nodularia douglasiae*. Phylogenetic relationships among 38 unionids based on the three following concatenated data sets: (1) an amino acid sequence alignment set (3259 aa) of 12 PCGs, (2) a full length nucleotide sequence alignment set (11,199 nt) of 12 PCGs and two rRNA genes, and (3) a reduced nucleotide sequence alignment set (7943 nt) of 12 PCGs except for 3rd codon position and two rRNA genes. The branch supporting values on each node are indicated in following order: (1) Bayesian posterior probability based on full length nucleotide sequence alignment, (2) Bayesian posterior probability based on reduced nucleotide sequence alignment except for 3rd codon position, (3) Bayesian posterior probability based on amino acid sequence alignment, (4) bootstrapping values of a maximum-likelihood tree based on full length nucleotide sequence alignment, (5) bootstrapping values of a maximum-likelihood tree based on reduced nucleotide sequence alignment except for 3rd codon position, and (6) bootstrapping values of a maximum-likelihood tree based on amino acid sequence alignment. *Maximum value (BI =1.00 and ML =100). #Case that all six different confidence values on a certain node appear maximums. The Korean *N. douglasiae* mitochondrial genome obtained from the present study is shown in bold letter on the tree. The three species belonging to the subfamily Margaritiferidae were employed as outgroups in this study.
